# Convergent construction of N-terminally modified CCL5 chemokines for photoaffinity receptor pull-down using cross-aldol bioconjugations

**DOI:** 10.1039/d5cb00162e

**Published:** 2025-09-18

**Authors:** Afzaal Tufail, Matthew E. Warnes, Nathalie Signoret, Martin A. Fascione

**Affiliations:** a Department of Chemistry, University of York, Heslington York YO10 5DD UK martin.fascione@york.ac.uk; b Experimental Medicine and Biomedicine Group, Hull York Medical School, University of York YO10 5DD UK; c York Biomedical Research Institute, University of York, Heslington York YO10 5DD UK nathalie.signoret@york.ac.uk

## Abstract

Chemokines such as CCL5 (RANTES) mediate immune responses *via* interaction with G-protein-coupled receptors like CCR5, which also serves as a co-receptor for HIV-1 entry into host cells. Modified CCL5 analogues have shown promise as CCR5 antagonists for anti-HIV strategies, but current approaches involve hydrolytically unstable linkages or laborious synthesis. Here, we demonstrate the use of an organocatalyst-mediated protein aldol ligation (OPAL) to construct N-terminally modified CCL5 analogues bearing hydrolytically stable carbon–carbon linkages. Using a recombinant CCL5 P2G mutant and selective oxidation to introduce an α-oxo aldehyde at the N-terminus, we achieved efficient OPAL bioconjugation with various aldehyde donors, including alkyl and aryl acetaldehydes. Notably, a 4-azido aryl acetaldehyde CCL5 OPAL product was utilised as a CCR5 photoaffinity probe. This modified chemokine successfully captured CCR5 from mammalian cells *via* photo-crosslinking, enabling receptor pull-down for biochemical analysis. Our work showcases cross-aldol bioconjugations as a versatile and convergent strategy for stable chemokine functionalisation, with potential applications in therapeutic development and mechanistic studies of chemokine–receptor interactions. This method offers a promising chemical biology platform for modulating or probing the CCL5–CCR5 axis with enhanced precision and synthetic accessibility.

## Abstract 2

Herein we demonstrate the use of an organocatalyst-mediated protein aldol ligation (OPAL) to construct modified CCL5 chemokines bearing a photoaffinity handle which can be used to pull-down CCR5 receptors from mammalian cells.

## Graphical abstract

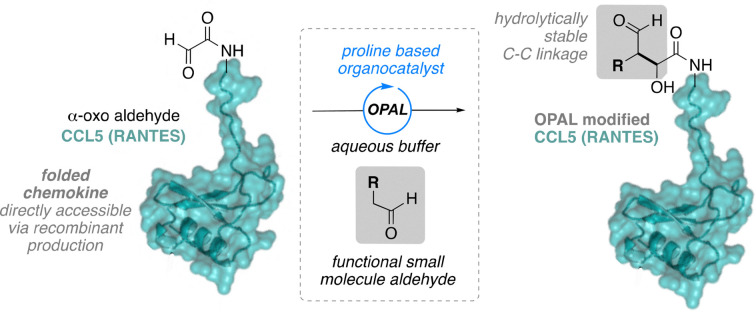

## Introduction

**Fig. 1:**
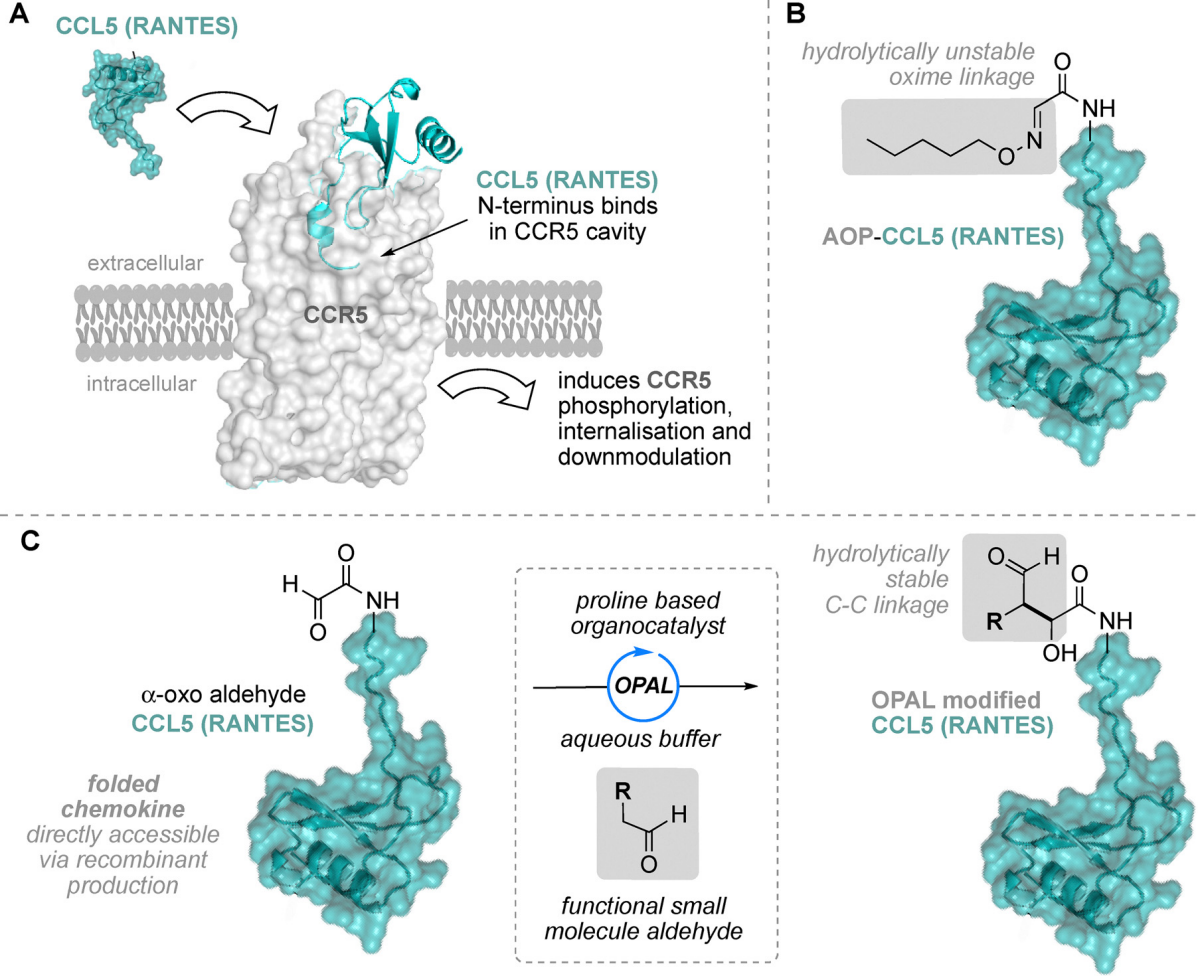
(A) CCL5 interaction with the extracellular cavity of CCR5 triggers a proinflammatory signaling cascade and the downmodulation of the receptor from the cell surface. (B) AOP-CCL5 (AOP-RANTES) is modified at the N-terminus with a hydrolysable oxime linkage. (C) Our convergent OPAL approach to modification of the CCL5 N-terminus *via* cross-aldol bioconjugation.

Chemokines are small proteins (8–14 kDa) that contain characteristic disulfides and mediate a range of functional biological processes including directed cell migration (chemotaxis) and activation induced-gene expression and proliferation^1–4^ through interaction with cell-surface chemokine G-protein coupled receptors. Human C–C chemokine ligand 5 (CCL5, also known as RANTES-regulated on activation, normal T cell expressed and secreted) is an agonistic ligand for the CCR5 chemokine receptor^5^ (Fig. 1A), and CCL5-mediated activation of CCR5 triggers a proinflammatory intracellular signalling cascade leading to the recruitment of immune cells to sites of infection and inflammation.^6,7^ However, CCR5 has also been implicated in cancer progression and Human Immunodeficiency Virus-1 (HIV-1) immune cell entry, whereby HIV-1 can bind to both CD4 and CCR5, as a co-receptor on target cells.^5^ Notably, native CCL5 has demonstrated potential in blocking HIV-1 infection^8^ by inducing CCR5 internalisation and downmodulation^9^ stimulating further research into modified chemokines. N-terminally modified CCL5 analogues have led the way in this field of therapeutic CCR5 blockade, but also represent an area of underexplored potential as chemical biology tools for studying the CCL5–CCR5 interactome.

Early N-terminally modified CCL5 derivatives developed included AOP-RANTES, which displayed potent sub-nanomolar HIV inhibitory activity^10^ acting as a super-agonist inducing sustained CCR5 phosphorylation and down-modulation.^11,12^ AOP-RANTES is modified through an oxime bond at the chemokine N-terminus, which binds in the CCR5 cavity, and is therefore a target for modulating the CCL5–CCR5 interaction (Fig. 1A). Further exploration of the N-terminal region also led to the development of PSC-RANTES,^13^ which displayed increased potency compared to AOP-RANTES but is constructed *via* total chemical synthesis of the chemokine, which is both laborious and expensive. Notably AOP-RANTES occupied CCR5 is subjected to rounds of endocytosis and recycling,^14^ whilst PSC-RANTES sequesters CCR5 within the cell.^15^ Other later approaches to generate CCL5-analogues utilised less laborious late stage convergent synthesis, where a synthesised CCL5 without the native N-terminus can be differentially elaborated with N-terminal pharmacophores.^16^ But this strategy primarily relies on the formation of carbon–heteroatom linkages including oximes, as in AOP-RANTES (CCL5) (Fig. 1B) and hydrazones, both of which have subsequently been shown to be hydrolytically unstable *in vivo*.^17,18^ Thus new convergent bioconjugation approaches that enable access to CCL5 analogues with hydrolytically stable linkages are still highly attractive, and their potential applications as chemical tools for further mechanistic study of the CCL5–CCR5 axis ripe for exploration. Our contribution to this area stems from the development of organocatalyst-mediated protein aldol ligation (OPAL) bioconjugations,^19^ which generate stable carbon–carbon linkages through cross-aldol coupling^20^ of small molecule aldehyde donors and proteins bearing reactive α-oxo aldehyde acceptors.^21^ Herein we demonstrate that the OPAL can be deployed in the convergent construction of N-terminally functionalised CCL5 analogues, using α-oxo aldehyde CCL5, accessible from recombinant protein and small molecule aldehyde probes (Fig. 1C). As the N-terminus of CCL5 embeds deeply into the extracellular cavity of its CCR5 receptor^22,23^ this method constitutes a platform approach for modulating or ‘capturing’ CCL5–CCR5 interactions at the cell surface, and we also showcase this utility in the construction of a CCL5-photoaffinity probe that enables selective CCR5 pull-down from mammalian cells.

## Results and discussion

**Fig. 2:**
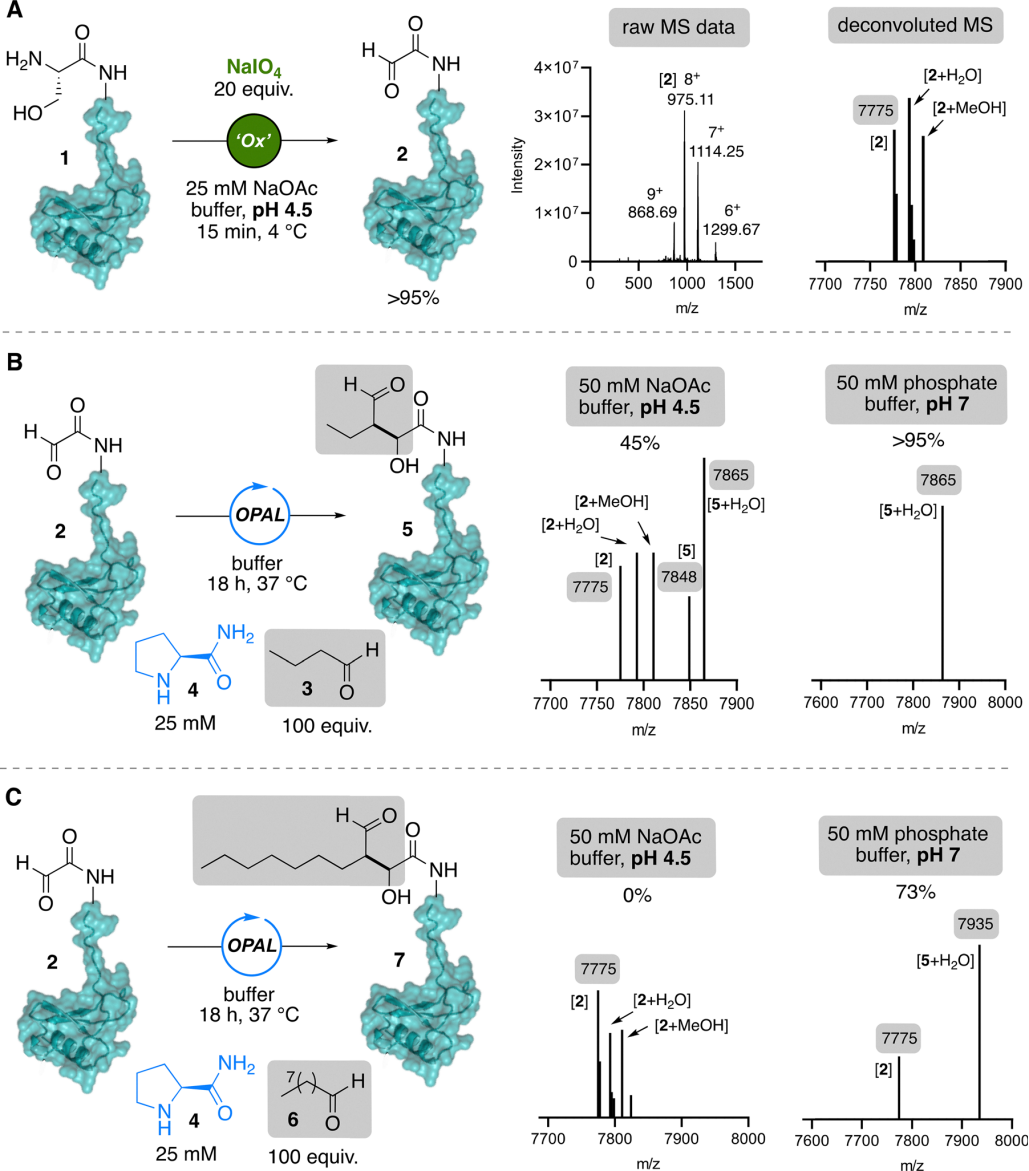
(A) Optimised NaIO_4_ oxidation of CCL5 P2G 1 with (right) raw and deconvoluted protein MS analysis (deconvoluted spectrum shows mass in Da/1) demonstrating the presence of species consistent with oxidised-CCL5 P2G 2. (B) OPAL modification of 2 with butyraldehyde 3 and prolinamide organocatalyst 4 and (right) deconvoluted protein MS analysis of reactions at pH 4.5 or pH 7 with starting material 2 and/or product 5 present. (C) OPAL modification of 2 with nonanal 6 and prolinamide organocatalyst 4 and (right) deconvoluted protein MS analysis (spectra show mass in Da/1) of reactions at pH 4.5 or pH 7 with starting material 2 and/or product 7 present.

To facilitate studies on the convergent N-terminal bioconjugation of CCL5 we needed easy access to the chemokine that did not involve total synthesis or low-yielding *in vitro* refolding of chemokine from inclusion bodies. We therefore opted to utilise our recently published method for recombinant expression of His-SUMO-CCL5 fusion protein,^24^ which following traceless cleavage of the SUMO tag under non-denaturing conditions affords soluble fully folded CCL5 with its native N-terminal serine residue. The presence of the N-terminal serine potentially enables facile access *via* periodate oxidation to the reactive N-terminal α-oxo aldehyde^25^ CCL5 (Fig. 1C), which is required as the electrophilic partner in our cross-aldol OPAL bioconjugation. Although highly reactive, N-terminal α-oxo aldehydes can undergo unproductive intramolecular cyclisation when the directly adjacent residue is a proline,^26,27^ as is the case in CCL5. Therefore, to maximise the stability of the aldehyde before bioconjugation in this study we used the CCL5 point mutant (P2G). The recombinant CCL5 P2G was purified at 28 mg L^−1^ from *E. coli* SHuffle LysY cells, used to aid folding of the two structural disufides that are required for chemokine function. Subsequent His-SUMO tag capture and SUMO protease cleavage afforded untagged CCL5 P2G 1 ready for screening in N-terminal periodate oxidation studies directly on the folded protein. Notably, CCL5 P2G was most amenable to reproducible periodate oxidation in acidic conditions to yield CCL5 bearing an α-oxo aldehyde handle 2 for OPAL modifications, as at neutral pH we observed that the protein would often precipitate at higher concentration, likely a consequence of CCL5 well-established proclivity for self-association^28,29^ which we observed to be both buffer pH and concentration dependant, consistent with previous studies demonstrating CCL5 can form large aggregates at high μM concentrations at pH 7.^30^ Under the optimised conditions at pH 4.5 in NaOAc buffer oxidation reached complete conversion by protein mass spectrometry (MS) within 15 min at 4 °C using a 20-fold excess of periodate (Fig. 2A).

Next, we set out to establish optimised OPAL conditions for modification of CCL5 P2G α-oxo aldehyde. Cognisant that the introduction of alkyl chains at the CCL5 N-terminus had previously yielded chemokines with non-native behaviour for CCR5 interactions, we initially attempted N-terminal OPAL bioconjugation using butyraldehyde 3 as the small molecule aldehyde donor. Following incubation with 100 equivalents of donor at pH 4.5 for 18 h in the presence of 25 mM prolinamide 4 as an organocatalyst, protein MS analysis demonstrated OPAL product 5 formation on the P2G mutant with 45% conversion (Fig. 2B), with wild-type CCL5 notably showing no turnover (Fig. S1), presumably due to the anticipated intramolecular cyclisation onto the aldehyde. Although the chemokine is less prone to oligomerisation at acidic pH, analogous bioconjugation at pH 7 significantly increased conversion to >95% product, demonstrating the OPAL reaction of CCL5 is more efficient under neutral conditions. Notably, reversible self-association could be limited by performing the OPAL at low μM CCL5 concentration at pH 7, followed by dialysis into lower pH with dissociation of the CCL5 into its active form. The presence of long alkyl chains at the N-terminus of CCL5 has afforded derivatives which can behave as super agonists, such as PSC-RANTES with bears a nine carbon N-terminal alkyl chain. We therefore also attempted to directly conjugate nonanal (9-carbon alkyl aldehyde) 6 to the α-oxo handle on the N-terminus of CCL5 P2G 2, and observed no conversion to product at pH 4.5, but 73% conversion to OPAL product 7 at pH 7 by protein MS when again using 100 equivalent of donor and prolinamide 4 as the organocatalyst (Fig. 2C). This unoptimized conversion using a long alkyl aldehyde, with poor aqueous solubility, is highly promising and suggests the OPAL bioconjugation does have potential as a platform approach to directly access a suite of alkyl derivatised chemokines for screening in receptors binding and activation experiments, which are ongoing in our group for report in due course.

**Fig. 3:**
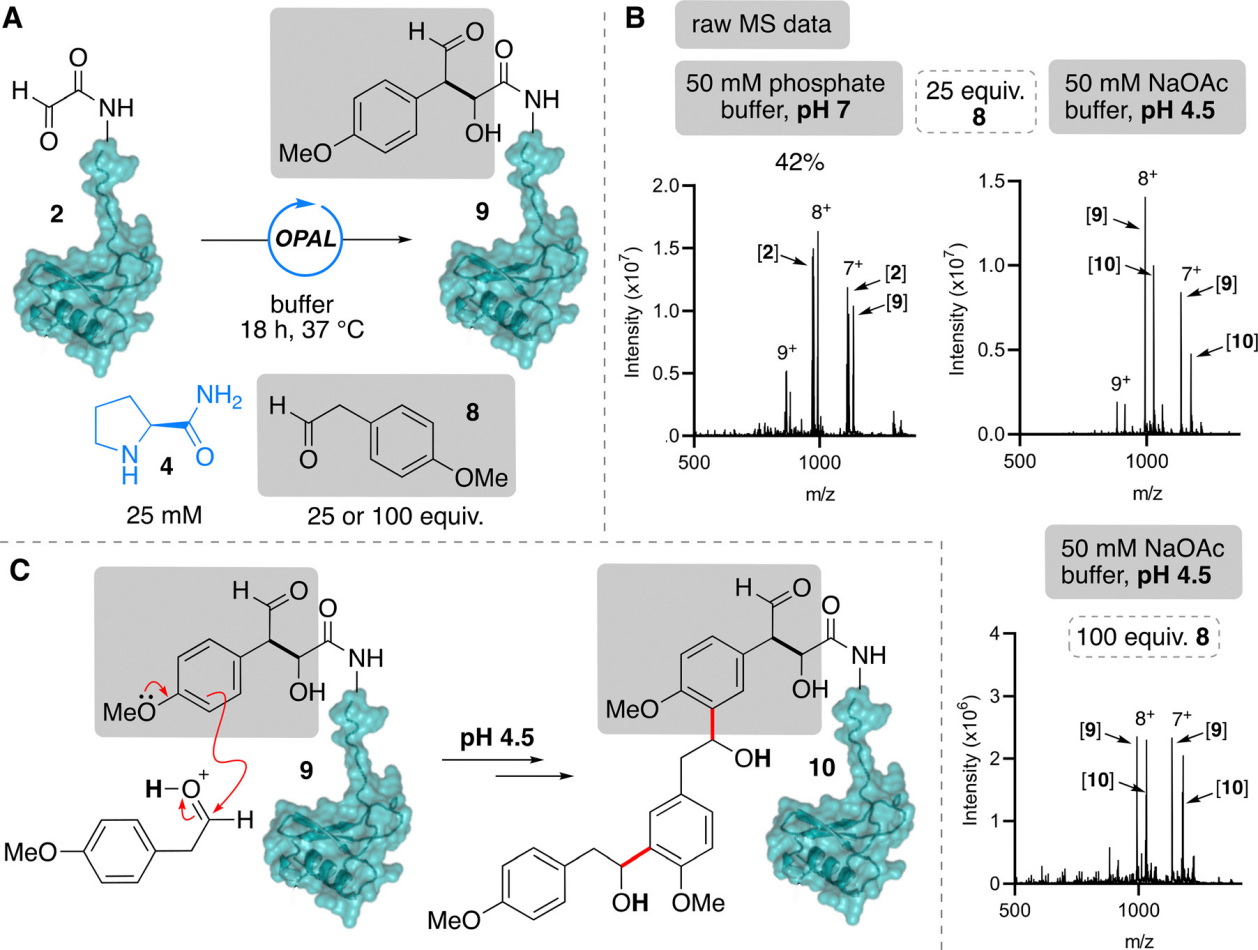
(A) Outline of OPAL modification of 2 with 4-methoxy phenylacetaldehyde 8 and prolinamide organocatalyst 4. (B) Raw protein MS analysis of reactions using 25 and 100 equiv. of 8 at pH 4.5 or pH 7 with starting material 2 showing formation of single OPAL addition product 9 and/or multiple additions product 10. (C) Proposed structure of multiple addition product 10 and mechanism for its formation under acidic conditions.

**Fig. 4:**
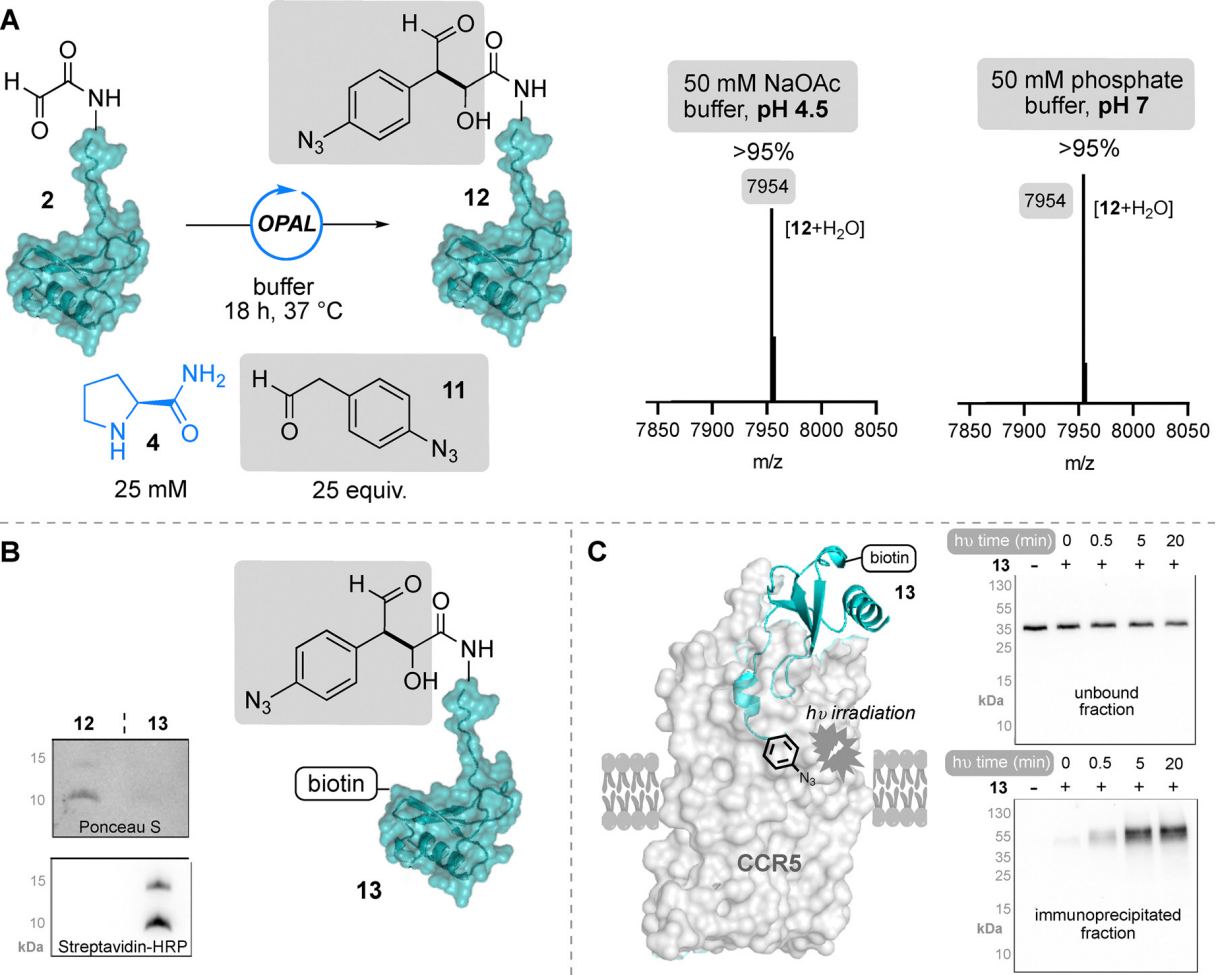
(A) OPAL modification of 2 with 4-azido phenylacetaldehyde 11 and prolinamide organocatalyst 4 and (right) deconvoluted protein MS analysis (spectra show mass in Da/1) of reactions at pH 4.5 or pH 7 with starting material 2 (and/or product 12 present. (B) Western blot analysis of bifunctional biotinylated aryl azide CCL5-P2G 13 with biotin visualisation by streptavidin-HRP chemiluminescence, note the potential presence of well-established CCL5 oligomerisation^29^ at 15 kDa. (C) Photo-crosslinking and immunoprecipitation of CCR5 on CHO-CCR5 cells with analysis of unbound fraction and immunoprecipitated fraction using anti-CCR5 MC5 antibody, following incubation with 13 and UV irradiation (365 nm).

In our previous studies, we demonstrated that aryl acetaldehydes showed even greater reactivity in cross-aldol bioconjugations than alkyl aldehydes, and the aromatic scaffold presents opportunity for facile introduction of further chemical functionality. We therefore also screened 4-methoxy phenylacetaldehyde 8 as an OPAL donor, using prolinamide 4 as an organocatalyst, in reaction with α-oxo-CCL5 P2G 2 at both acidic and neutral pH (Fig. 3A). Interestingly, at pH 4.5 with 25 equivalents of the electron rich 4-methoxy phenylacetaldehyde 8 (Fig. 3B, top right), we observed full conversion to species with masses consistent with single 9 and multiple additions 10 of the aldol donor, suggestive of formation of the OPAL product 9 followed by a subsequent condensations between the newly appended electron rich aromatic ring on the protein and the protonated 4-methoxy phenylacetaldehyde 8 now acting as an aldol acceptor (Fig. 3C). Increasing the 4-methoxy phenylacetaldehyde 8 to 100 equivalents led to increased conversion to the multiple additions product 10 (Fig. 3B, bottom right), whilst increasing the pH to 7 eradicated the multiple addition product, affording 42% conversion to only the OPAL product 9 (Fig. 3B, top left), thus reinforcing the hypothesis that multiple addition is driven by protonation of the small molecule. To circumvent this unwanted side reaction, we then utilised the less electron rich 4-azido aryl acetaldehyde 11 donor for OPAL modification of α-oxo-CCL5 P2G 2 (Fig. 4). As anticipated, using a 25 fold excess of donor and 25 mM prolinamide 4 as an organocatalyst, at both pH 7 and pH 4.5 we observed >95% conversion to the OPAL product 12 (Fig. 4A), with no multiple addition product, underlining the importance of considering the electronics of the aryl ring when using the OPAL reaction in acidic conditions.

Notably aryl azides are well established as chemically stable handles that can be activated by irradiation with light to generate highly reactive intermediates^31,32^ that have been widely used for protein crosslinking and profiling.^33^ Therefore, we sought to exploit the potential of the appended aryl azide functionality for capturing the non-covalent CCL5–CCR5 interaction by protein–protein photo-crosslinking, stabilising the cell surface interaction for downstream immunoprecipitation or “pull-down” of the receptor. As small amounts of CCL5 P2G precipitation were observed in OPAL reactions at pH 7, large scale OPAL bioconjugation with the 4-azido phenylacetaldehyde donor 11 was performed at pH 4.5 before non-selective biotin NHS-coupling performed to afford the bifunctional CCL5 P2G 13, confirmed by western blot with streptavidin-HRP mediated chemiluminescent visualization (Fig. 4B). CCR5 pull-down using the biotinylated aryl azide CCL5 P2G 13 was then attempted on a Chinese hamster ovary cell line (CHO), stably expressing CCR5.^11^ The chemokine photoaffinity probe was first prebound to CCR5 presenting cells, before the chemokine-receptor interactions were stabilised through photo-crosslinking at 365 nm for varying lengths of time ranging from 0.5–20 minutes. The cells were lysed and supernatants containing cytosolic proteins were incubated with streptavidin magnetic beads and the resulting unbound supernatant fraction collected for later analysis. Following washing, any protein bound to the beads were eluted and subjected to western blot alongside the unbound supernatant fraction. Monomeric CCR5 (∼36 kDa) was detected in the unbound fractions using an anti-CCR5 MC5 antibody western blot (Fig. 4C), with a slight band shift observed for CCR5 when comparing the no chemokine treatment control to samples treated with the CCL5 probe, indicative of CCR5 phosphorylation following activation by the ligand.^14,24^ Whilst the biotin pull-down elution fractions contained CCR5–CCL5 complex (∼50 kDa) primarily in samples that had been exposed to chemokine probe and irradiated with UV light with an irradiation-time dependent increase in protein concentration, and only low level ‘pull down’ of the CCL5–CCR5 complex in the absence of photo-crosslinking.

## Conclusions

In conclusion these experiments unequivocally demonstrated the capability of our N-terminally modified aryl azide chemokine to capture transient ligand-receptor interactions on mammalian cells. Although our approach requires the use of a CCL5 P2G variant to facilitate the OPAL reaction, we showed this mutant was still able to bind the receptor, albeit with a 3 fold reduction compared to wild-type CCL5 (Fig. S14), and that the mutant and its modified biotinylated aryl azide CCL5 P2G 13 both trigger characteristic phosphorylation of CCR5 Ser 349 and subsequent receptor endocytosis^6^ (Fig. S15). This exemplifies that chemokines modified under mild OPAL conditions retain functional biological activity and highlights the potential utility of cross-aldol OPAL as an efficient bioconjugation for exploring the chemokine ligand-receptor interactome, augmenting the existing chemokine chemical biology toolbox.

## Author contributions

Afzaal Tufail: conceptualization, investigation, methodology, visualization, formal analysis, writing – original draft. Mathew Warnes: investigation. Nathalie Signoret: funding acquisition, supervision, conceptualization, writing – original draft, review and editing. Martin A. Fascione: funding acquisition, supervision, conceptualization, writing – original draft, review and editing.

## Conflicts of interest

The authors declare no competing financial interest.

## Supplementary Material

CB-OLF-D5CB00162E-s001

## Data Availability

The data supporting this article have been included as part of the supplementary information (SI). Supplementary information is available. See DOI: https://doi.org/10.1039/d5cb00162e.
