# Supplementary material for: Convergent construction of N-terminally modified CCL5 chemokines for photoaffinity receptor pull-down using cross-aldol bioconjugations
Source: RSC Chem Biol. 2025 Sep 18;6(11):1772–8. doi: 10.1039/d5cb00162e (PMC12455665; doi:10.1039/d5cb00162e)
Supplement: CB-006-D5CB00162E-s001 [file CB-006-D5CB00162E-s001.pdf]

Supplementary Information (SI) for RSC Chemical Biology.
This journal is © The Royal Society of Chemistry 2025

# *Supplementary Information*

## Convergent construction of N-terminally modified CCL5 chemokines for photoaffinity receptor pull-down using cross-aldol bioconjugations

Afzaal Tufail[1,2], Matthew E. Warnes[1], Nathalie Signoret[2,3]* and Martin A. Fascione[1,3]*

[1]Department of Chemistry, University of York, Heslington, York, YO10 5DD, UK. [2]Experimental Medicine and Biomedicine Group, Hull York Medical School, University of York, YO10 5DD. [3]York Biomedical Research Institute, University of York, Heslington, York, YO10 5DD, UK.

**Supplementary Figures**

1. Challenges of CCL5 modifications – OPAL bioconjugation with butyraldehyde **3**
2. CCL5 P2G OPAL bioconjugation with 100 equiv. 4-methoxy phenylacetaldehyde **8** at pH 7.0
3. CCL5 P2G 1 h OPAL bioconjugation with 4-azido phenylacetaldehyde **11**
4. Full-length immunoblots
5. Mass spectrometry data
6. Biological activity of CCL5 P2G and the biotinylated aryl azide CCL5 P2G

## Methods

### Site-directed mutagenesis

The N-terminal Proline residue at position 2 in CCL5 was mutated for a glycine (P2G) in cDNA from the His-SUMO-CCL5 construct[1] using QuikChange Lightning Site-Directed mutagenesis kit (Agilent) and a pair of primers with the following sequence: Forward 5'-GAACAGATTGGTGGTAGCGGGTATAGCGATACCAC-3' and Reverse 5'-GTGGTATCGCTGCTATACCCGCTACCACCAATCTGTTC-3'. PCR reaction was carried out using 10 ng of plasmid DNA template and amplification cycling parameters detailed below (Table 1) to generate the His-Sumo-CCL5 P2G mutated DNA, before degrading the remaining DNA template using the restriction endonuclease Dpn 1 (37°C, 5 minutes). XL10-gold ultracompetent cells were transformed with the plasmid containing the His-Sumo-CCL5 P2G DNA, transformants selected with ampicillin (100 µg/mL), and positive colonies grown for miniprep DNA extraction.

| Segment | Cycles | Time | Temperature |
|---|---|---|---|
| 1 | 1 | 2 minutes | 95°C |
| 2 | 18 | 20 seconds | 95°C |
| | | 10 seconds | 60°C |
| | | 30 seconds/kb plasmid length | 68°C |
| 3 | | 5 minutes | 68°C |

Table 1. Cycling parameters for site-directed mutagenesis

### Purification of CCL5 P2G

Protein purification method was based on purification of IH(in house)-CCL5.[1] The Human CCL5 inserted sequence corresponds to the Uniprot sequence ID P13501 with a P2G mutant without the signal peptide.[2] In brief, His-Sumo-CCL5 P2G DNA was transformed in Shuffle LysY cells and positive colonies were used to inoculate a 1 L culture at 30°C (2xYT). Cultures were induced using 0.2 mM IPTG at $OD_{600}$ 0.7 and incubated at 30 °C for 5 h. Cells were sonicated in buffer A (80 mM Tris pH 8.2, 0.5 M NaCl & 30 mM imidazole) with protease inhibitors and lysates were clarified by centrifugation at 38,000 x g, 45 mins at 4 °C. Lysate was applied on an 5 mL FF Crude HisTrap column for initial his purification pre-equilibrated with buffer A. The column was washed with 10 column volumes of buffer A followed by a gradient over 6 column volumes to 100% buffer B (80 mM Tris pH 8.2, 0.5 M NaCl, 500 mM imidazole). The eluate was dialysed into 80 mM Tris pH 8.2, 0.5 M NaCl, 10% glycerol overnight at 4°C overnight. The dialysed protein was incubated with SUMO protease ULP1 (1:25) at 4 °C with mild agitation for 24 h. The cleaved protein was loaded onto a 5 ml HisTrap FF crude column pre-equilibrated with buffer AB (80 mM Tris pH 8.2, 0.5 M NaCl, 10 mM Imidazole). The column was washed with 6 column volumes of buffer AB followed by a step gradient to 100% buffer B for 5 column volumes. SDS-PAGE was used to determine the purity of the fractions. The protein was dialysed in sodium acetate pH 4.5 and 500 mM NaCl overnight, in 1% acetic acid overnight, in 0.1% TFA overnight and then lyophilised (stored at -80 °C). The yield of the purification was 28 mg/L of bacterial growth.

## LC-MS method

Mass Spectrometry of samples was performed using an UltiMate® 3000 photodiode array detector probing at 250–400 nm, coupled to a HCT ultra ETD II (Bruker Daltonics) ion trap spectrometer, using Chromeleon® 6.80 SR12 software (ThermoScientific), esquireControl version 6.2, Build 62.24 software (Bruker Daltonics), and Bruker compass HyStar 3.2-SR2, HyStar version 3.2, Build 44 software (Bruker Daltonics) at the Centre of Excellence in Mass Spectrometry, York. All mass spectrometry was conducted in positive ion mode. Protein samples were analysed in 1:1 water:acetonitrile + 1% (v/v) formic acid. Protein samples were analysed without the use of a column at a flow rate of 0.25 mL min−1 at RT. Samples were in water with 0.1% (v/v) formic acid (solvent A) and acetonitrile with 0.1% (v/v) formic acid (solvent B) mobile phase at a flow rate of 0.25 mL min−1 at RT.

## Mild periodate oxidation of CCL5 P2G

*N*-terminal serine oxidation of CCL5 P2G was carried out by resuspending desired amounts of lyophilised protein into 25 mM NaOAc pH 4.5 followed by the addition of 20 equiv. $NaIO_4$. The reaction was incubated at 4°C on ice (dark) after which the reaction was quenched with the addition of ethylene glycol. Reaction was buffered exchanged using PD SpinTrap G25 column into either LC-MS water for LC-MS analysis or 50 mM NaOAc pH4.5 for storage at -80°C. Mass spectrometry was used to assess the oxidation state of the protein.

## Organocatalyst protein aldol ligation of oxidised CCL5 P2G

An aliquot of oxidised CCL5 P2G in 0.05 M NaOAc pH 4.5 was charged with 0.5 M NaOAc pH 4.5 (0.05 M final concentration) and DMSO (5% final concentration). The reaction was then charged with s-prolinamide organocatalyst from a 1 M stock solution (0.025 M final concentration) and respective equiv. of aldehyde donor (1 M stock in DMSO). Reaction volume was completed to desired volume with LC-MS water and the solution was vortexed and allowed to sit at 37 °C for indicated time with agitation (350 rpm). Conversion to the aldol products were confirmed by LC-MS.

## Synthesis of 4-azido phenylacetaldehyde 11

4-azido phenylacetaldehyde **11** was synthesised as described in previous work.[3]

## Biotinylation of 4-azido phenylacetaldehyde linked CCL5 P2G

Thermo Scientific antibody biotinylation kit for IP (cat. 90407) was used to biotinylate the azido linked CCL5 P2G. PD SpinTrap G25 column was equilibrated with 1x PBS pH 7.2 for buffer exchange. Lyophilised protein was resuspended in PBS pH 7.2 (180.6 µM) and combined with, 1x PBS and NHS-PEG4-Biotin at 40-fold molar excess was combined to a final volume of 180 µL and allowed to react for 0.5 h at RT. Following this, the reaction was buffer exchanged into 1x PBS. Biotinylated protein sample at 1 µg of protein per lane were run on a 15% SDS-PAGE gel and transferred onto nitrocellulose. Membrane were stained with Ponceau S and probed with streptavidin-HRP and HRP signal was visualised on iBright CL1500 software version 1.8.0.

**Immunoprecipitation**

Thermo Scientific IP-MS kit (cat. 90408) was used for the immunoprecipitation assays. 5 million cells per condition were used and treated as indicated. Briefly, CHO-CCR5 cells were detached using 10 mM EDTA-PBS and resuspend in Binding Medium (BM: RPMI 1640 without carbonate or glutamine, 0.2% (w/v) BSA, 10 mM HEPES adjusted to pH 7) at $2x10^6$/mL. Cells were treated with 30 nM modified CCL5 P2G for 1 h at RT. Cells were washed 1x with BM prior to photo crosslinking of the azido motif linked to the CCL5 P2G protein using 365 nm light (10 cm) for the indicated time after which cells were lysed at $1x10^7$ cells/mL for 10 minutes on ice in presence of protease inhibitors and immunoprecipitation performed using Thermo-Scientific IP-MS kit (). Cell debris were removed by centrifugation at 13,000 x g and supernatants applied onto washed magnetic streptavidin beads for 4 h RT with agitation. Unbound protein was removed for analysis by western blot and beads were washed according to the manufacturer recommendations. Bound protein was eluted off the beads by boiling with 1x reducing sample buffer (95°C, 10 mins). Samples were run on a 15% SDS-PAGE gel and transferred onto a nitrocellulose membrane that was blocked in 5% milk and probed with anti-CCR5 antibody MC5 (1 µg/mL) O/N.[1] Membranes were washed with 1x TBS-T, incubated with anti-mouse HRP (1/5000, Sigma-Aldrich cat. A9044) 1 h at RT and chemiluminescence detection signal was visualised on iBright CL1500 software version 1.8.0.

## Supplementary Figure 1.

Challenges of IH-CCL5 modifications

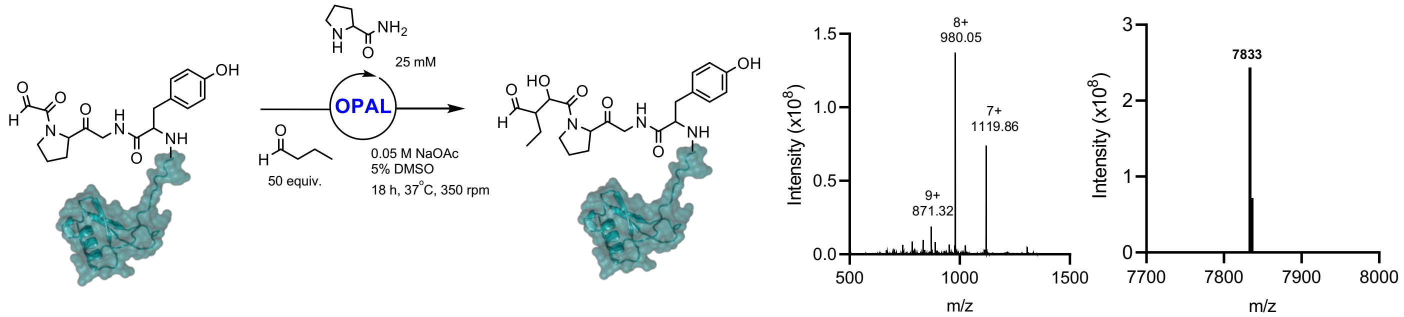


**Figure S1. OPAL reaction of oxidised IH-CCL5 with butyraldehyde 3**
OPAL reaction was attempted on oxidised IH-CCL5. Reaction proceeded for 18 h at 37°C and conversion was assessed using mass spectrometry analysis. Mass of oxidised IH-CCL5 (7834 Da – hydrate) and OPAL product (7888 Da).

## Supplementary Figure 2.

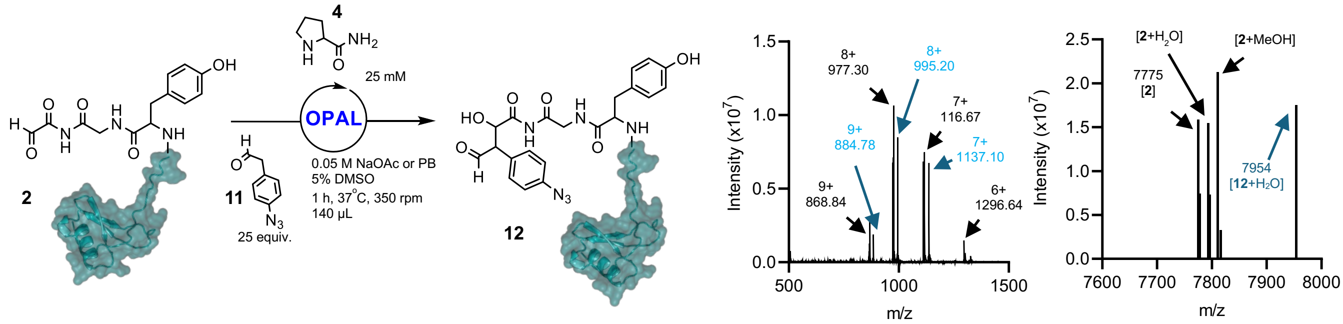


**Figure S2. Small scale 4-azido phenylacetaldehyde 11 OPAL with oxidised CCL5 P2G 1 h**
4-azido phenylacetaldehyde **11** was conjugated onto oxidised CCL5 P2G using OPAL chemistry in acidic pH. Reaction proceeded for 1 h at 37°C and conversion was assessed using mass spectrometry analysis.

**Supplementary Figure 3.**

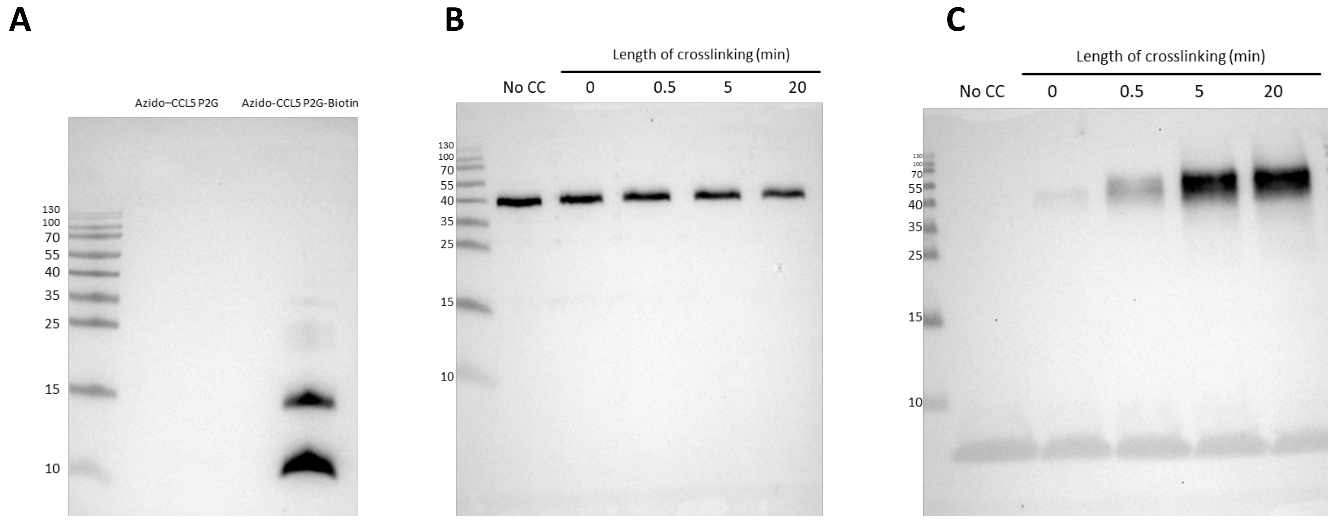


**Figure S3. Full-length immunoblots**

(A). Full-length immunoblot for the biotinylation of CCL5 P2G coupled with 4-azido phenylacetaldehyde **11** and biotin. (B) Full-length gel for the unbound fraction IP and (C) Full-length gel for the IP elution. (A) Immunoblot with streptavidin-HRP and (B/C) immunoblot with anti-CCR5 MC5 antibody (1 μg/mL).

## Mass spectrometry data

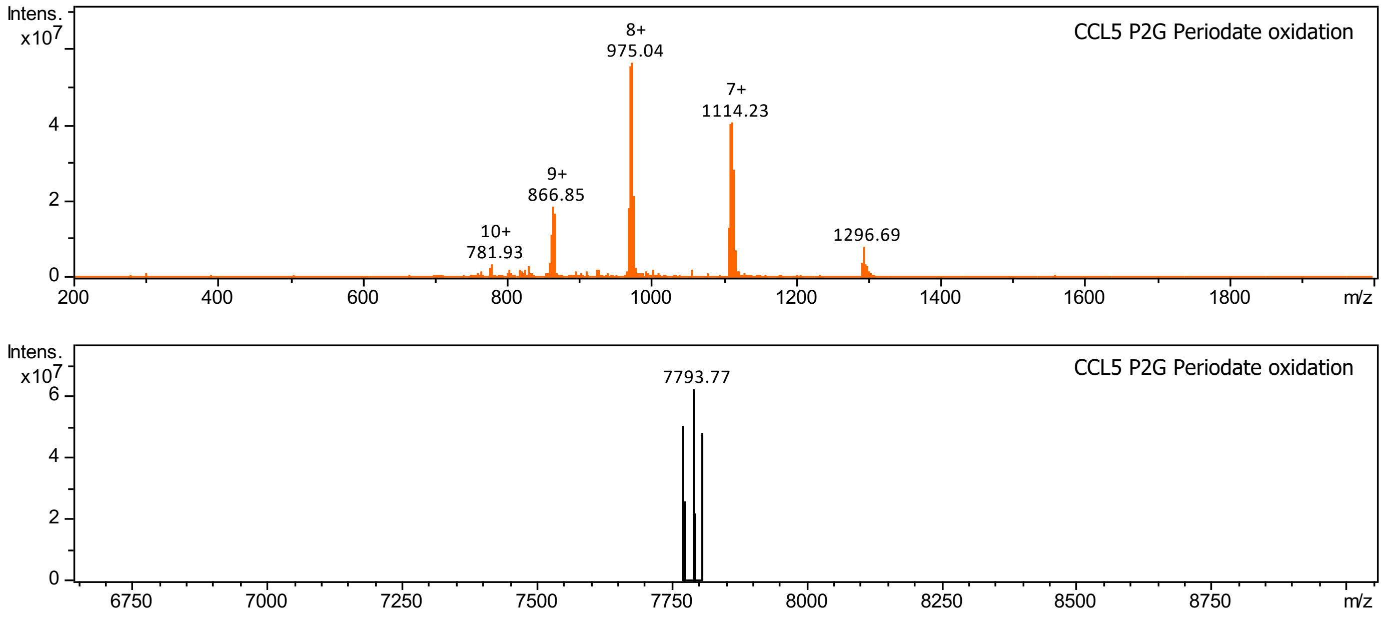


**Figure S4. Mass spectrometry analysis following NaOAc oxidation of CCL5 P2G**
$NaIO_4$ oxidation of CCL5 P2G **1** with protein MS demonstrating the presence of species consistent with oxidised-CCL5 P2G **2** (calculated – 7775 Da; observed – 7775 Da).

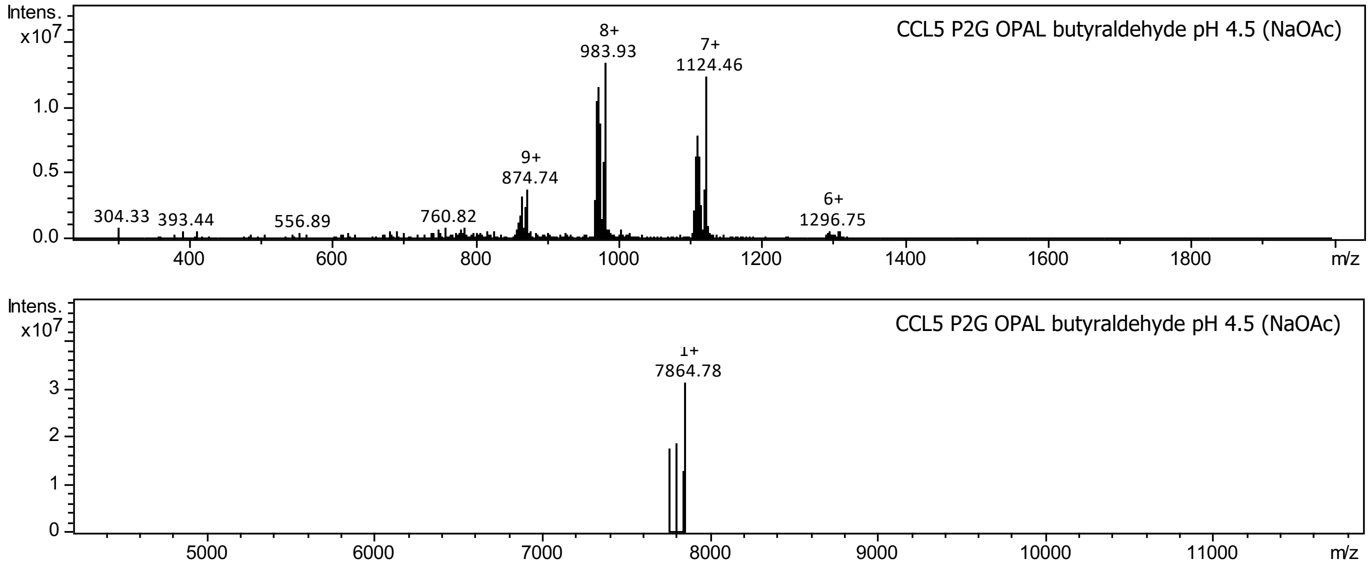


**Figure S5. Mass spectrometry analysis following OPAL bioconjugation of CCL5 P2G 2 with butyraldehyde 3 at pH 4.5 NaOA**
OPAL modification of **2** with butyraldehyde **3** at pH 4.5 with starting material **2** (calculated – 7775 Da; observed - 7775 Da) and product **5** present (calculated – 7865 Da (+$H_2O$); observed – 7865 Da)

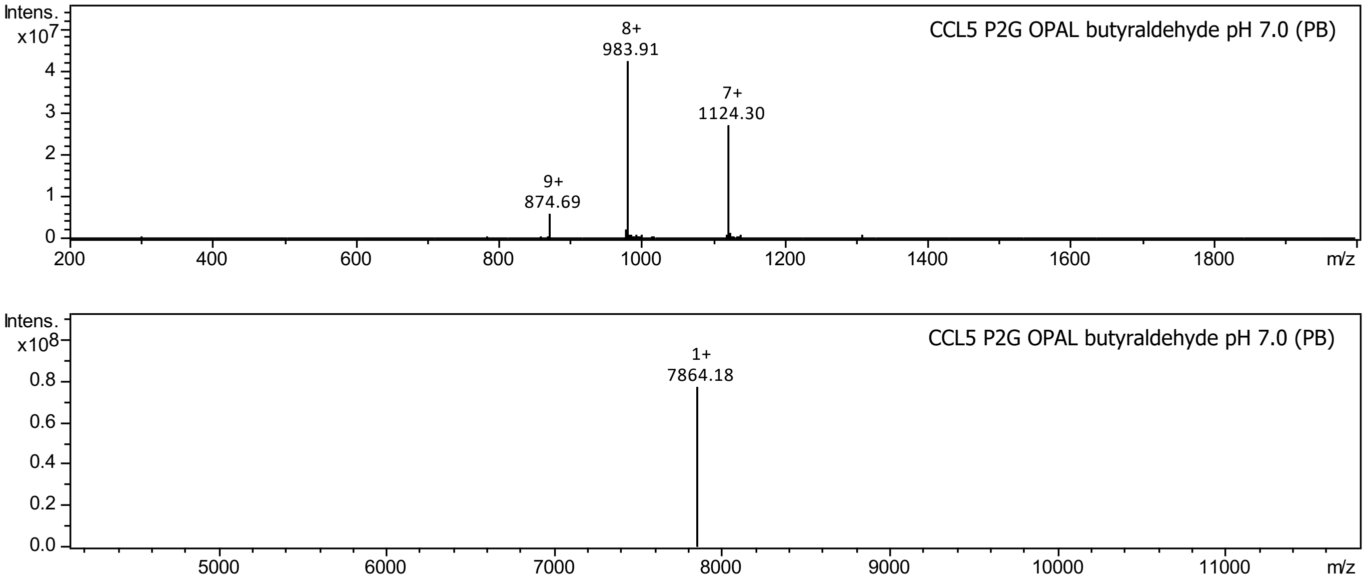


**Figure S6. Mass spectrometry analysis following OPAL bioconjugation of CCL5 P2G 2 with butyraldehyde 3 at pH 7.0 PB**

OPAL modification of **2** with butyraldehyde **3** at pH 7.0 with product **5** present (calculated – 7865 Da (+$H_2O$); observed – 7865 Da)

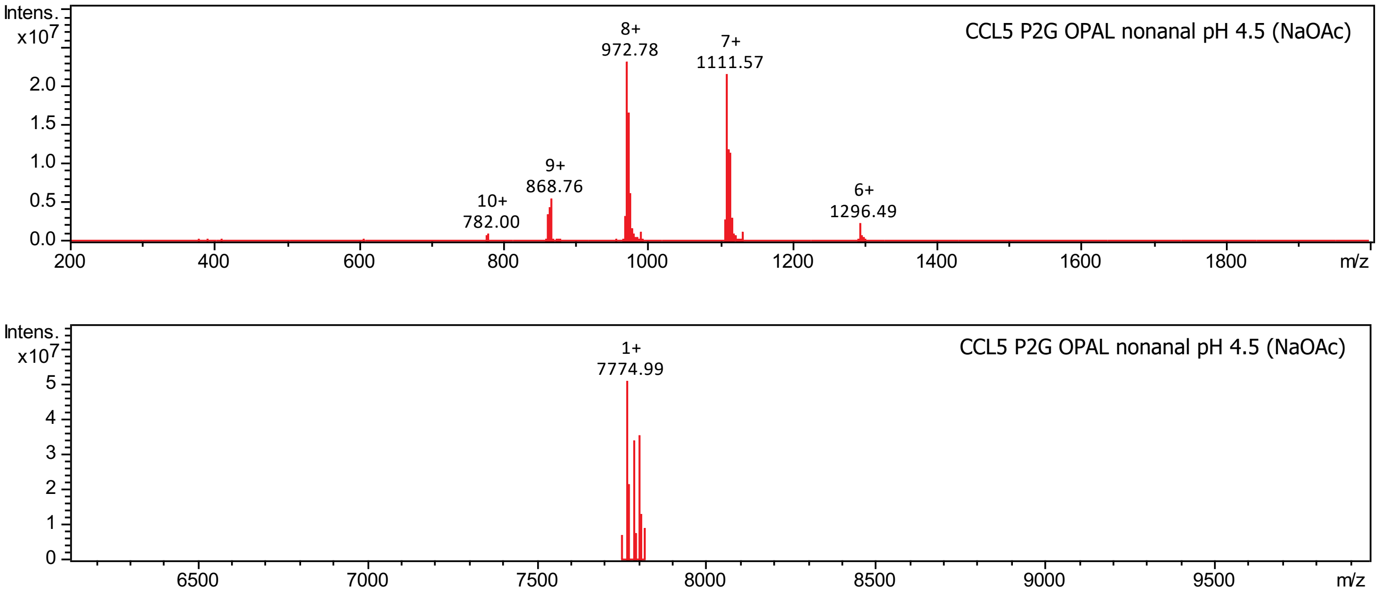


**Figure S7. Mass spectrometry analysis following OPAL bioconjugation of CCL5 P2G 2 with nonanal 6 at pH 4.5 NaOAc**

OPAL modification of **2** with nonanal **6** at pH 4.5 with only starting material **2** present following reaction (calculated – 7775 Da; observed - 7775 Da).

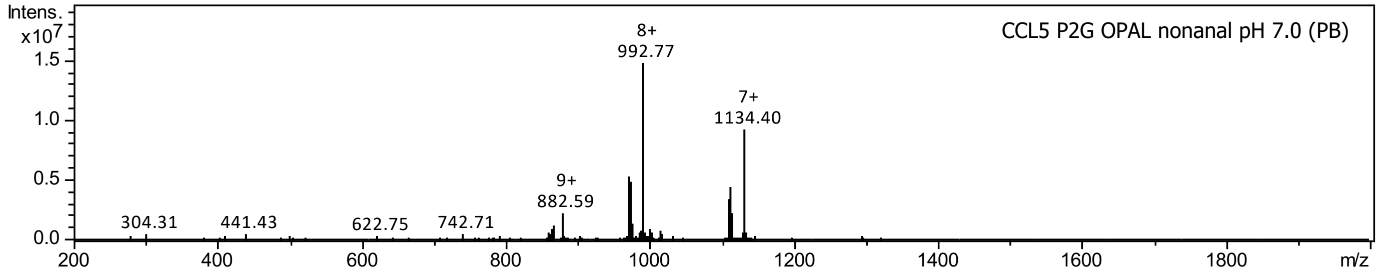


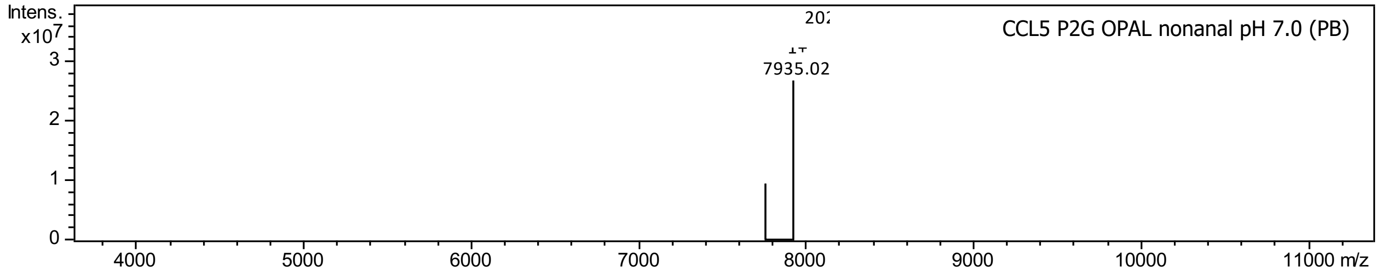


**Figure S8. Mass spectrometry analysis following OPAL bioconjugation of CCL5 P2G 2 with nonanal 6 at pH 7.0 PB**

OPAL modification of **2** with nonanal **6** at pH 7.0 with starting material **2** (calculated – 7775 Da; observed - 7775 Da) and product **7** (calculated – 7935 Da (+$H_2O$); observed – 7935 Da).

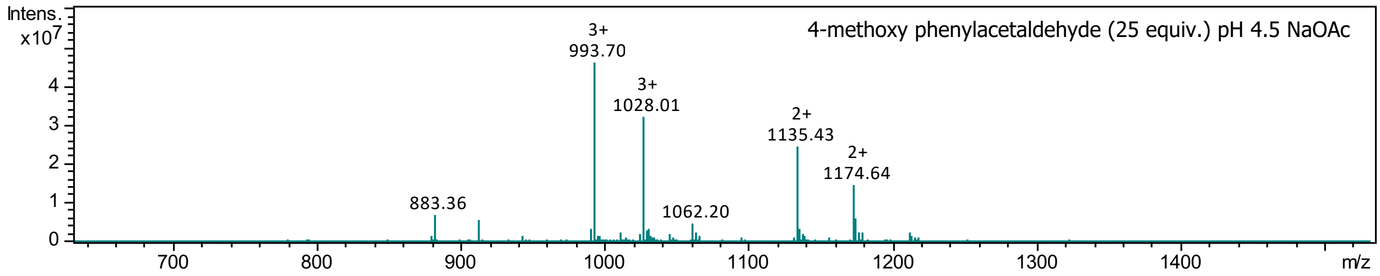


**Figure S9. Mass spectrometry analysis following OPAL bioconjugation of CCL5 P2G 2 with 25 equiv. 4-methoxy phenylacetaldehyde 8 at pH 4.5 NaOAc**

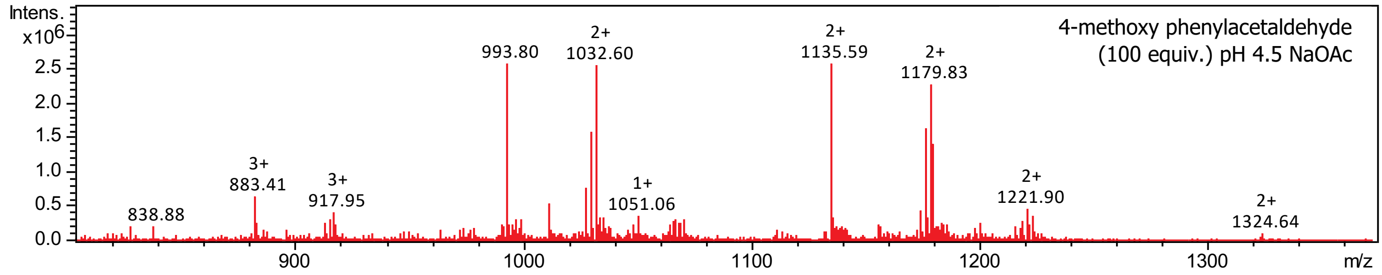


**Figure S10. Mass spectrometry analysis following OPAL bioconjugation of CCL5 P2G 2 with 100 equiv. 4-methoxy phenylacetaldehyde 8 at pH 4.5 NaOAc**

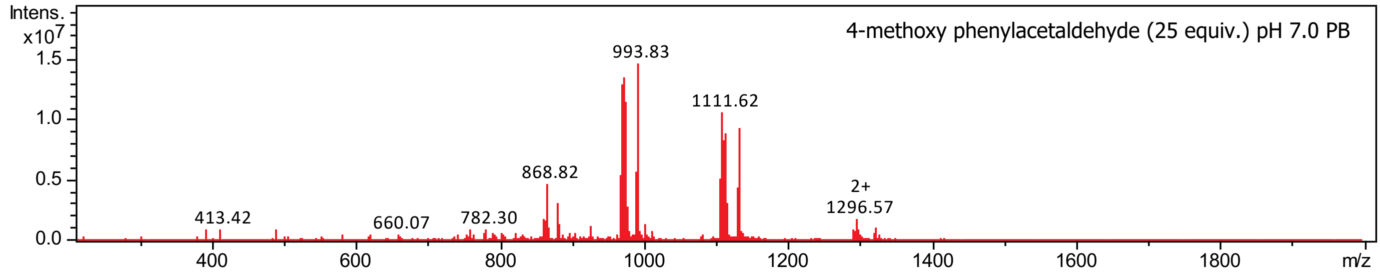


**Figure S11. Mass spectrometry analysis following OPAL bioconjugation of CCL5 P2G 2 with 25 equiv. 4-methoxy phenylacetaldehyde 8 at pH 7.0 PB**

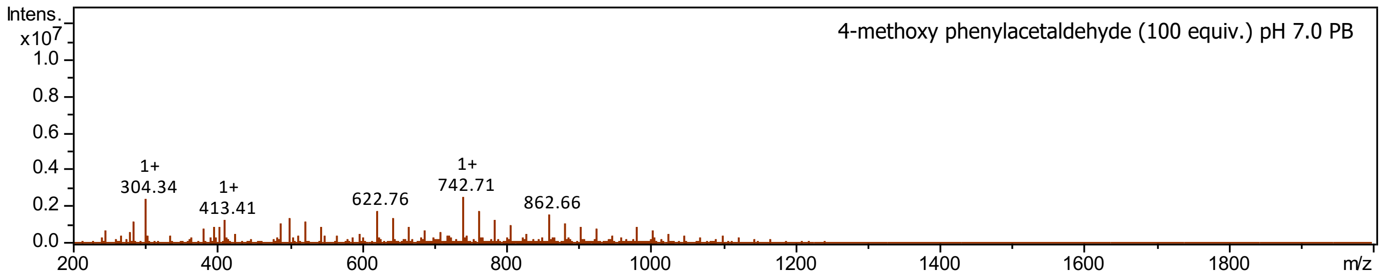


**Figure S12. Mass spectrometry analysis following OPAL bioconjugation of CCL5 P2G 2 with 100 equiv. 4-methoxyphenylacetaldehdye 8 at pH 7.0 PB**

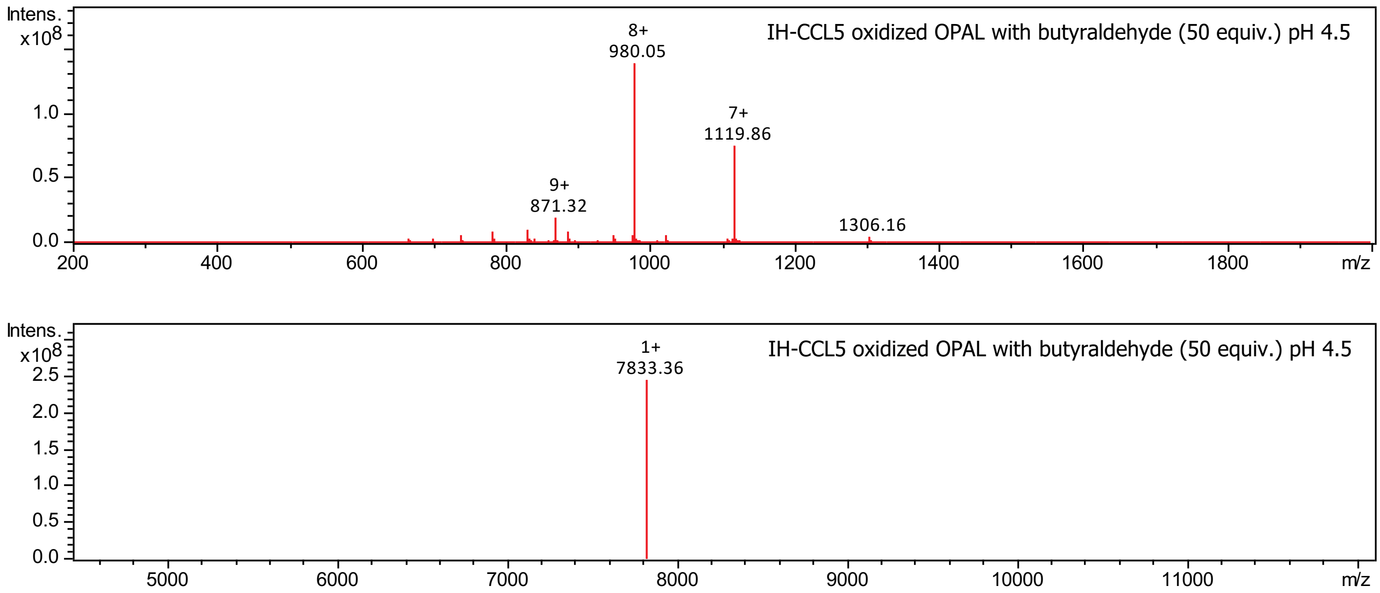


**Figure S13. OPAL reaction of oxidised IH-CCL5 with butyraldehyde 3**
OPAL reaction was attempted on oxidised IH-CCL5. Reaction proceeded for 18 h at 37°C and conversion was assessed using mass spectrometry analysis with only starting material oxidised IH-CCL5 (calculated – 7834 Da (+$H_2O$); observed - 7834 Da).

## Biological activity of CCL5 P2G and the biotinylated aryl azide CCL5 P2G

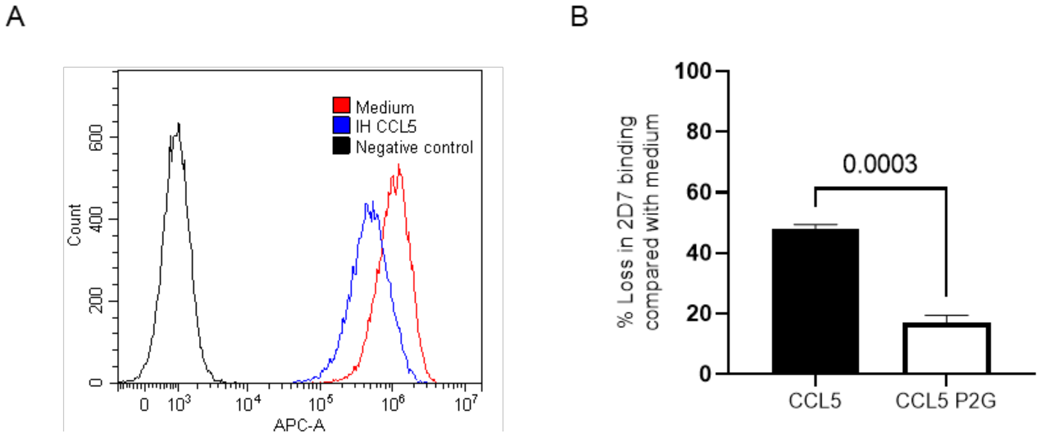


**Figure S14. Detection of CCL5 P2G binding to CCR5 using indirect masking approach.**
(A) Anti-CCR5 2D7 antibody binding epitope overlaps with CCR5 chemokine binding site[4] resulting in reduced cell-associated fluorescence after CCL5 binding to CHO-CCR5 measured by flow cytometry. (B) Loss in 2D7 mAb binding compared with medium. Cell treatment with 100 nM of CCL5 or CCL5 P2G at RT was followed by fixation and cells labelling with 5 ug/mL of 2D7 for 1 h at 4°C detected with secondary anti-mouse A647 (4 ug/mL). T-test statistical analysis with Welch's test.

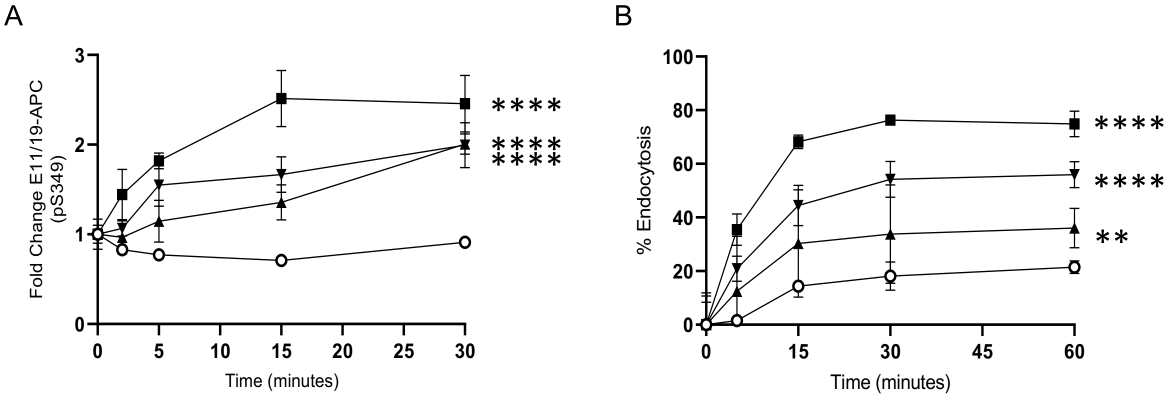


**Figure S15. Biotinylated aryl azide CCL5 P2G induced CCR5 phosphorylation and endocytosis.** Ligand activation of CCR5 leads to GRK mediated CCR5 phosphorylation at serine-349 and clathrin mediated receptor endocytosis. (A) Kinetic of CCR5 phosphorylation using phospho-FLOW and (B) Kinetics of ligand induced CCR5 endocytosis. Two-way Anova compared with medium with Dunnett test. **** <0.0001 and ** 0.0021. Ligands included -o- medium, -■- IH-CCL5, -▲- CCL5 P2G and -▼- biotinylated aryl azide CCL5 P2G.

## References:


1. A. Tufail, S. Akkad, A. R. Noble, M. A. Fascione and N. Signoret, *Sci. Rep.*, 2024, **14**, 24188.
2. T. J. Schall, J. Jongstra, B. J. Dyer, J. Jorgensen, C. Clayberger, M. M. Davis and A. M. Krensky, J. Immunol. Baltim. Md 1950 141, 1018–1025
3. N. D. J. Yates, S. Akkad, A. Noble, T. Keenan, N. E. Hatton, N. Signoret and M. A. Fascione, *Green Chem.*, 2022, **24**, 8046-8053.
4. L. Wu, G. LaRosa, N. Kassam, C. J. Gordon, H. Heath, N. Ruffing, H. Chen, J. Humblias, M. Samson, M. Parmentier, J. P. Moore, C. R. Mackay, *J. Exp. Med.*, 1997, **186**(8):1373-81
